# Analysis of Health Insurance Big Data for Early Detection of Disabilities: Algorithm Development and Validation

**DOI:** 10.2196/19679

**Published:** 2020-11-23

**Authors:** Seung-Hyun Jeong, Tae Rim Lee, Jung Bae Kang, Mun-Taek Choi

**Affiliations:** 1 Sungkyunkwan University Suwon Republic of Korea; 2 Korea Disabled People's Development Institute Seoul Republic of Korea

**Keywords:** early detection of disabilities, health insurance, big data, feature selection, classification

## Abstract

**Background:**

Early detection of childhood developmental delays is very important for the treatment of disabilities.

**Objective:**

To investigate the possibility of detecting childhood developmental delays leading to disabilities before clinical registration by analyzing big data from a health insurance database.

**Methods:**

In this study, the data from children, individuals aged up to 13 years (n=2412), from the Sample Cohort 2.0 DB of the Korea National Health Insurance Service were organized by age range. Using 6 categories (having no disability, having a physical disability, having a brain lesion, having a visual impairment, having a hearing impairment, and having other conditions), features were selected in the order of importance with a tree-based model. We used multiple classification algorithms to find the best model for each age range. The earliest age range with clinically significant performance showed the age at which conditions can be detected early.

**Results:**

The disability detection model showed that it was possible to detect disabilities with significant accuracy even at the age of 4 years, about a year earlier than the mean diagnostic age of 4.99 years.

**Conclusions:**

Using big data analysis, we discovered the possibility of detecting disabilities earlier than clinical diagnoses, which would allow us to take appropriate action to prevent disabilities.

## Introduction

Providing intervention support early by detecting a child's risk factors for disability helps to prevent not only the disability itself, but also secondary disability by eliminating the risk factors [1-8]. When detection is delayed, the risk of developmental delay is also increased, as the child is unable to perform developmental tasks. If a child's disability is detected after 6 years of age, the child has passed the optimal period of language development, which leads to difficulties in language communication [9].

The main reasons for delayed detection are initial perception by parents, the physician’s wish to delay diagnosis until the prognosis is clearer, or a mistaken assumption by parents that the disorder will improve [3,10]. Childhood developmental delays are difficult to diagnose from a single symptom, as there is a possibility that a temporary delay in development is erroneously considered a disability. If there is no intervention, due to a delay in the detection of the risk of disability in infants and toddlers, the prognosis may not be good [5].

In order to detect a child's disability early, parents must recognize the indications early and request related assistance; policy should provide support to make this possible. However, there is a limit to policy that expands support for assessment costs and reach. Public awareness and education that enables parents to recognize disabilities early should be implemented, but also, in the long run, a system should be established in which the government can identify risk factors in children even if parents do not recognize them early. It is, therefore, necessary that institutions, such as daycare centers and hospitals, are trained to detect risk factors of disability as soon as possible and to provide parents with relevant information.

Utilizing health insurance big data for early detection may open many possibilities. In South Korea, a system of compulsory medical insurance benefits was initiated in 1977 under the National Health Insurance Act; more than 97% of the public now have obligatory medical insurance, and all related data, including those on diseases and health, are kept and managed by the Korea National Health Insurance Service (KNHIS) [11,12]. With the enactment of the Elderly Long-Term Care Insurance Act in 2007, information relating to the health, nursing, and medical care of older adults is gathered and stored in a cutting-edge information and communications technology database [13]. The data provided by KNHIS contain not only health care provider information but also vast amounts of data (about 2.1 trillion) from people’s birth to death [14].

Machine learning techniques that allow computer models to learn knowledge from data [15] can be used to analyze big data such as those in the sample cohort data from KNHIS. Since the medical insurance data contain physician diagnosis records for individuals, the information can be used to label the data, where it becomes a supervised learning problem [16]. Moreover, database classification is a type of supervised learning. It is a process of analyzing existing data to determine the class of newly observed data [17]. Problems that require classification into multiple classes are called multiclass problems.

With the recent availability of national health insurance big data for research purposes, relevant research has commenced. However, since the big data from KNHIS includes sensitive personal information, only some modified data can be used and analyzed through remote access to the KNHIS computer systems. When applying for data export, only deidentified analysis results are made available. Due to these limitations, big data analysis using the health insurance data is still in its infancy [18]. One study [19] that uses KNHIS big data analyzed the correlation between certain diseases, such as sinusitis surgery and asthma. Another study [20] identified diseases that were more likely to occur by using similar group-based data analysis to develop an app service that provides personalized disease and hospital information.

As far as we know, very little research has been done on developing a systematic approach to the early detection of disabilities using big data. Chang [21] examined a supervised learning method for early intervention in children with delayed development based on the clinical data of 516 children below 6 years of age. The study [21] analyzed the association between language, motor, social, and cognitive development from identified diseases, visual problems, psychological and intellectual development, other diseases, and types of delay and, using compositions of the decision tree, made 14 association rules derived scores support and confidence scores. David and Balakrishnan [22] applied a decision tree algorithm and rough sets for the prediction of learning disabilities in school-age children using a checklist of 16 most frequent signs and symptoms of learning disabilities (n=513, area under the receiver operating characteristic curve [AUROC] 0.985). Varol et al [23] present the application of machine learning methods for early prediction of reading disability, collecting 356 samples using 40 features, including demographics, pretesting, and weekly monitoring (word identification fluency); the comparison was made using 6 classification algorithms, and the best result was an AUROC of 0.942. Although these studies [21-23] have showed good learning results on specific disabilities, there are limitations in applying them to all disabilities; since the data used in these studies did not include lifelong records of people with disabilities, temporal tracking for early detection may not be feasible.

The purpose of this study was to detect risk factors for disabilities in children as early as possible based on medical data. Since we conducted early detection analysis on all disabilities, including delayed developmental disabilities, the results are likely to be more meaningful than those of previous studies. By analyzing the effect of each correlation, the disease that is the main cause of the disability could be identified. In this study, various classification algorithms were developed and optimized to find the best model for early detection. As it was based on KNHIS big data, it can lead to more in-depth studies of disabilities in the future.

Our research has the following novelties. As far as we know, it is the first time that a study has investigated early detection using comprehensive disability types using health insurance big data. In order to find the age at which the disability can be diagnosed early, we organized the data by age ranges and created an optimal classification model for each age range. We used multiclass classification algorithms to find the best model for each age range. The earliest age range with clinically significant performance shows the age at which disabilities can be detected early.

## Methods

### Data

We used medical data extracted from the KNHIS Sample Cohort 2.0 DB, which is an anonymized research database with information on health insurance qualifications, income, history of the hospital and clinic use, and results of health examinations and nursing institutions from 2002 to 2013, covering 1 million people (2% of Korea's 50 million people). Each sample in Sample Cohort 2.0 DB was labeled: no disability, physical disability, brain lesions, visual impairment, hearing impairment, and other disabilities. Other disabilities included all disability types such as speech disability, intellectual disability, and mental disorder. The database contains not only diagnostic codes based on the International Classification of Diseases (ICD) but also additional data such as prescription records, duration of treatments, and frequency of treatments. The distribution of the samples in Sample Cohort 2.0 DB is inherently imbalanced [11,24]. This study complies with the bioethics policy by the institutional review board of Korea National Institute for Bioethics Policy (P01-201905-22-005).

From the raw data, we selected samples for our analysis as follows. The samples we were able to collect at the time of analysis were records up to the age of 13 years, which would not be an issue for early detection. First, data were extracted from children with acquired disabilities with no missing records from birth to recorded diagnosis, which yielded 804 data records. We selected twice as many data records of children with disabilities, which yielded 1608 data records, to prevent the performance of our analytical model from being distorted by having the number records for those without disabilities being much more than the that of the records with disabilities.

Each sample was identified using a 7-digit personal identification number. Disease diagnostic data and prescription record data were extracted using personal identification numbers. Information on the date of medical treatment and diagnostic codes were available from the disease diagnostic data, classified using disease classification division codes. Prescription record data, such as the date and contents of prescriptions, were extracted from the records. Information on the number of medical actions and prescribed dosage was also recorded.

To discover the age at when the disabilities occurred, the medical records of each sample were organized in units of 1-year increments. The distribution of samples is shown in Table 1. Data for each age range were collected to construct a data set and used for classification learning. In order to improve stability and convergence speed during the optimization process, each feature was transformed to have a mean of 0 and a standard deviation of 1.

**Table 1:** Data samples by age range.

Age range (years)	No disability	Physical disability	Brain lesions	Visual impairment	Hearing impairment	Other disabilities	Total
Up to 1	1482	40	182	31	47	504	2286
Up to 2	1371	40	182	31	46	504	2174
Up to 3	1263	40	173	30	44	502	2052
Up to 4	1149	40	162	29	41	499	1920
Up to 5	1036	40	147	27	40	489	1779
Up to 6	935	38	137	23	35	473	1641
Up to 7	824	37	122	19	32	446	1480
Up to 8	714	27	102	17	27	400	1287
Up to 9	601	22	84	16	21	324	1068
Up to 10	478	21	70	14	18	265	866
Up to 11	363	18	59	11	15	199	665
Up to 12	242	14	39	6	9	134	444
Up to 13	123	6	17	2	3	71	222

### Feature Selection

Feature selection allows selection of a subset of relevant features [25,26]. Good feature selection can make models easier to interpret, shorten learning time, improve learning accuracy, and help avoid the curse of dimensionality [27,28]. We used the extra trees algorithm for feature selection, which is a method of randomly partitioning nodes using a candidate characteristic and then selecting the best partition among them, rather than finding an optimal threshold for partitioning nodes to generate a tree randomly [29]. For the implementation of feature selection, we used ExtraTreeClassifier (scikit-learn, version 0.23.1; Python, version 3.6) [30].

### Classification Algorithms

Since there are 6 categories in this study, it is a typical example of multiclass classification. We compared classification algorithms to develop the best model for the early detection of disabilities. We used 4 algorithms in this study: k-nearest neighbor, random forest, logistic regression, and gradient boosting.

The k-nearest neighbor algorithm finds *k* training data closest to the input and uses the output information of these data to estimate the output [31]. Small *k* values indicate a high risk of overfitting, while large values create boundaries with a high propensity to generalization. A variety of methods, such as Euclidean distance, Manhattan distance, and Mahalanobis distance [32], may be used to find adjacent data.

In the random forest model, predictions are generated by bagging several decision trees. Bagging is an ensemble meta-algorithm designed to improve stability and accuracy. Decision trees are similar to the game 20 questions; data are continuously separated based on the characteristics of the data, and the decision tree is classified into 1 correct answer [33,34].

Logistic regression is a linear model that predicts using linear combinations of independent variables [35]. Logistic regression estimates the probability for each group and classifies the data into a group according to a threshold, so it can be applied to the problem of classification [36].

Gradient boosting is a powerful learning algorithm that combines gradient descent with boosting. Gradient descent is an optimization method that reduces error by moving the error function in the opposite direction to the derivative. Boosting is a method that combines simple and weak learners to make more accurate and powerful learners [37,38]. Even if the accuracy is low, the model compensates for the calculated error [39].

### Model Learning

To verify the generalization performance of the model, we divided the data into training data (70%) and test data (30%). Training data were used to train the model; test data were used to evaluate the true classification performance of the trained model.

To find the best model for detecting disabilities, the 4 algorithms were trained. Each classification algorithm has hyperparameters, which when adjusted, show very different performances. Therefore, finding the optimal hyperparameter combination is necessary [30]. We used a grid search to find the optimal combination of hyperparameters for each algorithm. The model was checked against other data to avoid generalization errors during the grid-search process. We used 10-fold cross-validation to avoid further partitioning of data for validation. We used scikit-learn for all implementations.

### Performance Metrics

To specify indicators used to evaluate models in this study, we used confusion matrices such as Table 2. The confusion matrix is easy to visually identify when evaluating model performance [40].

**Table 2:** Confusion matrix for binary classification.

Actual	Predicted	
	Positive	Negative
Positive	True positive	False negative
Negative	False positive	True negative

Accuracy, the most common model performance indicator, is used to show how accurately the model predicts the input data. On the confusion matrix, accuracy is estimated by the sum of the true values divided by the whole; *accuracy* = (*true positive* + *true negative*) / *all*. Precision or the positive predictive value is an indicator of how accurately a model is able to predict a positive; *precision* = *true positive* / (*true positive* + *false positive*). Recall or sensitivity index is the ratio of actual values detected by the model to the actual values; *recall* = *true positive* / (*true positive* + *false negative*). If the data are unevenly distributed, accuracy can lead to distorted performance estimates. The F1 score expresses the harmonic mean of precision and recall. The F1 score gives equal importance to precision and recall. If the data are unevenly distributed, accuracy can lead to distorted performance estimates. Therefore, using F1 scores to measure performance allows for better performance comparisons than those using accuracy [41]; *F1 score* = 2 × *precision* × *recall* / (*precision* + *recall*). The weighted average method was used to measure the average of the indicators for each class; this method assigns a weight according to the number of samples. The weighted average is expressed by the following equation.

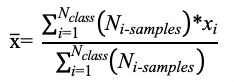



where 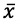 is the weighted average, *x_i_* is the result from the *i*th class, *N_class_* is the number of classes, and *N_i-samples_* is the number of samples in the *i*th class.

## Results

### Early Detection Using Only Disease Diagnostic Data

In our analysis using only ICD disease diagnostic data, we selected the top 150 out of the 4344 disease diagnosis features. Table 3 lists the 10 most important features.

**Table 3:** Top 10 features in terms of importance when using only disease diagnostic data.

Feature code	Feature name	Importance
F_	Mental and behavioral disorders	0.0498
I10	Essential (primary) hypertension	0.0327
I109	Unspecified hypertension	0.0161
G470	Disorders of initiating and maintaining sleep (insomnias)	0.0145
K259	Unspecified as acute or chronic gastric ulcer without hemorrhage or perforation	0.0133
K590	Constipation	0.0125
E785	Hyperlipidemia, unspecified	0.0120
M4806	Spinal stenosis, lumbar region	0.0120
K295	Chronic gastritis, unspecified	0.0119
J039	Acute tonsillitis, unspecified	0.0114

In model learning, the random forest algorithm performed best across all age ranges (results of the test data set are shown in Table 4). Our aim was to find the earliest age range with an F1 score close to or above 80% to ensure clinical significance [42]. Although the F1 score for up to 6 years was 83.4%, this was not meaningful because the average clinical diagnostic age was 4.99 years according to Sample Cohort 2.0 DB. Up to 4 years had an F1 score of 79.6%, which is close to 80%, and the age range is clinically meaningful. This model would detect disability almost 1 year earlier, given that the average clinical diagnostic age is 4.99 years.

**Table 4:** Model learning results when using only disease diagnostic data.

Age range (years)	Classifier	Parameters	Accuracy	Precision	Recall	F1 score
Up to 1	Random forest	n estimators: 16	0.703	0.639	0.703	0.660
Up to 2	Random forest	n estimators: 64	0.758	0.718	0.758	0.725
Up to 3	Random forest	n estimators: 64	0.800	0.778	0.800	0.776
Up to 4	Random forest	n estimators: 64	0.816	0.798	0.816	0.796
Up to 5	Random forest	n estimators: 64	0.818	0.787	0.818	0.796
Up to 6	Random forest	n estimators: 128	0.852	0.833	0.852	0.834
Up to 7	Random forest	n estimators: 64	0.836	0.805	0.836	0.813
Up to 8	Random forest	n estimators: 64	0.850	0.836	0.850	0.835
Up to 9	Random forest	n estimators: 128	0.854	0.837	0.854	0.838
Up to 10	Random forest	n estimators: 128	0.852	0.832	0.852	0.836
Up to 11	Random forest	n estimators: 64	0.873	0.854	0.873	0.856
Up to 12	Random forest	n estimators: 128	0.864	0.866	0.864	0.863
Up to 13	Random forest	n estimators: 64	0.922	0.929	0.922	0.914

The confusion matrix of the analysis for the range up to 4 years is given in Table 5. As the model was learned, the average for each class was high. Thus, the results of the confusion matrix indicate that most samples for children without disabilities were well classified.

**Table 5:** Confusion matrix when using only disease diagnostic data.

Actual	Predicted					
	No disability	Physical disability	Brain lesions	Visual impairment	Hearing impairment	Other disabilities
No disability	334	0	0	0	0	11
Physical disability	7	0	0	0	0	5
Brain lesions	4	0	34	0	0	10
Visual impairment	6	0	1	1	0	1
Hearing impairment	1	0	0	0	6	5
Other disabilities	46	0	9	0	0	95

### Early Detection Using Disease Diagnostic and Prescription Data

A second analysis was performed by adding prescription record data to the disease diagnostic data used in the previous analysis. Prescription data included information on medications, treatment materials, and medical practices received by patients. We used the top 150 out of a total of 12,713 features, including 4344 diseases and 8369 prescription data. Table 6 lists the 10 most important features.

**Table 6:** Top 10 features in terms of importance when using disease diagnostic and prescription data

Feature code	Feature name	Importance
F6203	Social Maturity Scale	0.0215
F_	Mental and behavioral disabilities	0.0124
F6201	Intelligence test	0.0123
NN011	Personal supportive psychotherapy	0.0105
F6215	Personality test (pictorial test)	0.0089
FY731	Childhood Autism Rating Scale	0.0087
NN031	Family therapy	0.0075
130801ASY	Hypnotic sedatives	0.0063
NN013	Personal intensive psychotherapy	0.0060
F6240	Bender Gestalt Test	0.0057

In model learning, both random forest and gradient boosting algorithms performed well (Table 7). In this analysis, the F1 score of the up to 4-year age range was 81.6%, which indicates that the early detection of disabilities seems to be relatively certain. In addition, as the F1 score for the up to 3-year age range was 78.3%, it is possible that improvements could lead to a diagnosis about 2 years before 4.99 years.

**Table 7:** Model learning results based on disease diagnostic and prescription data.

Age range (years)	Classifier	Parameters	Accuracy	Precision	Recall	F1 score
Up to 1	Logistic regression	*C*=0.1	0.732	0.691	0.732	0.688
Up to 2	Gradient boosting	learning rate: 0.4; n estimators: 4	0.767	0.743	0.767	0.738
Up to 3	Random forest	n estimators: 128	0.802	0.800	0.802	0.783
Up to 4	Random forest	n estimators: 128	0.832	0.819	0.832	0.816
Up to 5	Random forest	n estimators: 32	0.835	0.813	0.835	0.817
Up to 6	Gradient boosting	learning rate: 0.4; n estimators: 4	0.858	0.850	0.858	0.853
Up to 7	Random forest	n estimators: 32	0.849	0.830	0.849	0.834
Up to 8	Random forest	n estimators: 128	0.866	0.848	0.866	0.854
Up to 9	Gradient boosting	learning rate: 0.4; n estimators: 4	0.857	0.859	0.857	0.857
Up to 10	Random forest	n estimators: 128	0.898	0.878	0.898	0.885
Up to 11	Random forest	n estimators: 64	0.914	0.916	0.914	0.905
Up to 12	Gradient boosting	learning rate: 0.4; n estimators: 1	0.832	0.833	0.832	0.829
Up to 13	Gradient boosting	learning rate: 1.0; n estimators: 1	0.891	0.896	0.891	0.893

The confusion matrix of the analysis for the range up to 4 years is given in Table 8. As this was a learned model, the average for each class was high. The results of the confusion matrix, therefore, indicate that most children without disabilities were correctly classified. Children with physical disabilities were still not well classified, but there was some improvement in most classes.

**Table 8:** Confusion matrix when using disease diagnostic and prescription data.

Actual	Predicted					
	No disability	Physical disability	Brain lesions	Visual impairment	Hearing impairment	Other disabilities
No disability	336	0	0	0	0	9
Physical disability	6	0	5	0	0	1
Brain lesions	4	0	36	0	0	11
Visual impairment	4	0	1	4	0	1
Hearing impairment	3	0	0	0	5	4
Other disabilities	35	0	17	0	0	98

## Discussion

In this study, we used big data analysis for early detection of children who are more likely to have disabilities. An analysis of the sample data suggests that it is possible to detect disability early with accuracy at 3 or 4 years, which is before the average diagnostic age of 4.99 years. This means that children who may be at risk of disability due to various risk factors can be screened early based on medical records alone and can receive appropriate treatment to reduce the degree of disability.

The contributions of our study are described as follows. Our study is one of the first to investigate early detection of disabilities, covering all disabilities comprehensively based on KNHIS big data. This shows that health insurance data is of great value in analyzing disabilities and provides a basis for future studies. To find the age at which disabilities can be detected early, we set up a multiclass classification frame that organizes data by age ranges and trains multiple algorithms to select the best model. This frame can be further improved so that it could be an important tool for experts in the field.

Our study has the following limitations. Though it would be better if the disability was detected by age 3 years or earlier, the early detection performance from the up to 3-year age range did not exceed the clinically significant threshold of 80% due to limitations in health insurance sample data. Another limitation was that the other category of disabilities hampered the performance of the model. Future research with more data and detailed classification of other types of disabilities could lead to a more accurate analysis. The imbalance of samples also had an important impact on data analysis. In this analysis, the number of children with disabilities was 804; of which, 504 had other types of disabilities. Since data on physical disability, visual impairment, and hearing impairment were relatively less, the model may not have learned sufficiently; therefore, it is necessary to ensure that there is sufficient data for each type when conducting further studies. We chose the best model based on the F1 score, but in practice, depending on the situation, we may choose the best model with the least false positives or false negatives.

To improve the early detection model in the future, the following work can be done in the future. In addition to the records of diagnosed diseases and prescription medications used in this analysis, various data such as health medical examination data, are also collected by the National Health Insurance Service. Incorporating these additional data to overcome the abovementioned limitations could lead to the development of more sophisticated models for early disability detection analysis. Moreover, feature engineering is important because the number of features can increase tremendously, and future studies require a more diverse application and comparison of feature engineering algorithms.

## Abbreviations

AUROC: area under the receiver operating characteristic curve
ICD: International Classification of Diseases
KNHIS: the Korea National Health Insurance Service
